# N-acetyl cysteine through modulation of HDAC_2_ and GCN_5_ in the hippocampus mitigates behavioral disorders in the first and second generations of socially isolated mice

**DOI:** 10.1016/j.ibneur.2025.01.014

**Published:** 2025-01-25

**Authors:** Najmeh Asgharzadeh, Ali Noori Diziche, Hossein Amini-Khoei, Nasrin Yazdanpanahi, Mehrdad Shahrani Korrani

**Affiliations:** a Department of Biology, Falavarjan Branch, Islamic Azad University, Isfahan, Iran; b Medical Plants Research Center, Basic Health Sciences Institute, Shahrekord University of Medical Sciences, Shahrekord, Iran

**Keywords:** Open field, CA1, CA3, Morris water maze, Forced swimming

## Abstract

**Objective(s):**

Social isolation stress (SIS) in early life can lead to behavioral disorders. N-acetylcysteine (NAC), an antioxidant, may aid treatment. This study explored NAC's impact on behavior in first and second-generation mice after SIS, focusing on HDAC2 and GCN5 expression in the hippocampus.

**Materials and methods:**

In this study, 24 male and 24 female mice were bred for one generation. The pups were divided into six (3male, 3female) groups (n = 20): 1- Control receiving normal saline, 2- SIS with normal saline, 3- SIS with NAC (150 mg/kg) IP for four weeks. Eight mice from each group underwent behavioral, histopathological, and molecular tests, while others were mated (4 males + 4 females) to produce second generations. These pups were divided into 9 groups (n = 8) for behavioral tests, including elevated plus maze, open field, forced swimming, and histopathological and molecular assessments (HDAC2 and GCN5 expression) in the hippocampus.

**Results:**

The SIS group showed increased HDAC2 and GCN5 expression. Following SIS, there was a decrease in open arm entries and passes in the open field test, alongside increased immobility in the forced swimming test and reduced CA1 and CA3 hippocampal diameters. NAC mitigated the adverse molecular, behavioral, and histopathological impacts of SIS across both generations.

**Conclusion:**

NAC reduces behavioral disorders after SIS (first and second generation) by reducing the expression of GCN5 and HDAC2 and increasing neuronal diameter in the hippocampus. Future research should investigate the long-term therapeutic effects of NAC for behavioral disorders after SIS.

## 1 Introduction

Mental disorders are a group of disorders that affect the way of thinking and behavior of the affected person and cause disruption in the functioning of the patient. So far, the causes of these disorders have not been well identified. Genetic factors and different stresses in life can provide the basis for the occurrence of such disorders. Bipolar disorder, obsession, depression, anxiety, and schizophrenia are examples of mental disorders (Hsu et al., 2024).

Worldwide, one in eight people is affected by a mental disorder (Tahira, 2024). Epigenetic factors control the growth of genes that help brain development (Khosroshahi et al., 2024). Mental disorders increase the chance of developing other diseases such as cardiovascular diseases. Disturbance in the neuronal development of the hippocampus is one of the most important things that occur in this group of patients. Scientists have identified more than 270 parts of the human genome related to mental disorders (Zhu et al., 2024). Inhibition of HDAC_2_ (Histone deacetylase 2) gene expression improves brain damage (Yang et al., 2021). Early life stress causes very long-term epigenetic changes in the offspring's brain and increases the expression of the HDAC_2_ gene in the hippocampus region (Sun et al., 2021). Inhibition of HDAC_2_ increased long-term memory in laboratory mice and increased hippocampus function (Kyrke-Smith et al., 2021). General Control Non-repressed 5 protein (GCN_5_) is a histone acetyltransferase that stimulates the expression of some genes (Torres-Zelada and Weake, 2021).

GCN_5_ plays an extensive role in energy metabolism. The activator controls the transcription of energy-related genes (Mutlu and Puigserver, 2021). Scientists believe that acetylation can occur on a large number of proteins and regulate cellular functions (Albaugh and Denu, 2021). SIS leads to long-term behavioral disturbances linked to anxiety-like behaviors, including stress (Farzan et al., 2023). Being alone and socially isolated in children causes misbehavior during childhood and increases the risk of developing mental disorders in them (Zavitsanou et al., 2024). Also, SIS causes poor cognitive performance with anxiety behaviors (Yu et al., 2024).

NAC increases life expectancy. It is a precursor of glutathione and prevents the death of neurons. It has been determined that NAC attenuated behavioral disorders and exerted neuroprotection effects. Administration of NAC reduces the incidence of mental disorders (Zhang et al., 2024, Romero-Miguel et al., 2024). NAC during the development period of the nervous system prevents the disruption of the growth of neurons (Lai et al., 2022, Malik et al., 2023).

The aim of the researchers of this project is to investigate the effect of NAC on behavioral disorders in the first and second generations of mice subjected to SIS focusing on GCN_5_ and HDAC_2_ gene expression as well as CA_1_ and CA_3_ histological changes in the hippocampus.

In this study, first and second generation mice were used to investigate the epigenetic effects of social isolation.

## 2 Materials and methods

### 2.1 Ethics

All experiments were performed according to the institutional guidelines. Animal tests and all samplings were done according to the Falavarjan University Islamic Azad regulations (Ethical code: IR.IAU.FALA.REC.1401.020) and Ministry of Health guidelines regarding working with laboratory animals. This study was reported following the ARRIVE guidelines.

### 2.2 Animals

24 male NMRI mice were paired with 24 female NMRI mice (1 male and 1 female in each cage). Pregnant mice were kept in Plexiglas cages at a temperature of 22 ± 1 °C and a humidity of 50 ± 10 % under conditions of 12 h of light and 12 h of darkness. The initial weights of mice were 20–25 gr.

After birth, 60 female mice and 60 male mice were selected after three weeks of birth. After three weeks, the pups were divided into 6 groups (n = 20) including SIS and control were treated with normal saline (10 ml/kg) and NAC (sigma-Aldrich grade>99 %) (150 mg/kg) for 4 weeks. 1- The control group that received normal saline. 2- The group underwent SIS and received normal saline. 3- The group underwent SIS and received intraperitoneal NAC. 8 mice from each group were used for hippocampus histopathological examination (evaluation of CA_1_and CA_3_) and behavioral tests including (EPM, OFT, FST) and molecular evaluation of hippocampus (HDAC_2_ and GCN_5_ gene expression) (Romero-Miguel et al., 2024, Banos-Gomez et al., 2017).

First generation (Six groups):

(A) 20 female pups were subjected to SIS and received normal saline as a SIS group.
(B) 20 female pups received normal saline as a control group.
(C) 20 female pups subjected to SIS also received NAC.
(D) 20 male pups subjected to SIS and normal saline.
(E) 20 male pups were subjected to normal saline as a control group.
(F) 20 male pups subjected to SIS also received NAC.

And other mice were mated with each other to get the second generation. The second generation were divided into 9 groups (A, B, C, D, E, F, G, H, I) (n = 8) and were subjected to a behavioral test including (open field, EPM, forced swimming) histopathology test to assess CA1, CA3 diameter and molecular evaluation of gene expression (HDAC2 and GCN5).

**Fig. 1:**
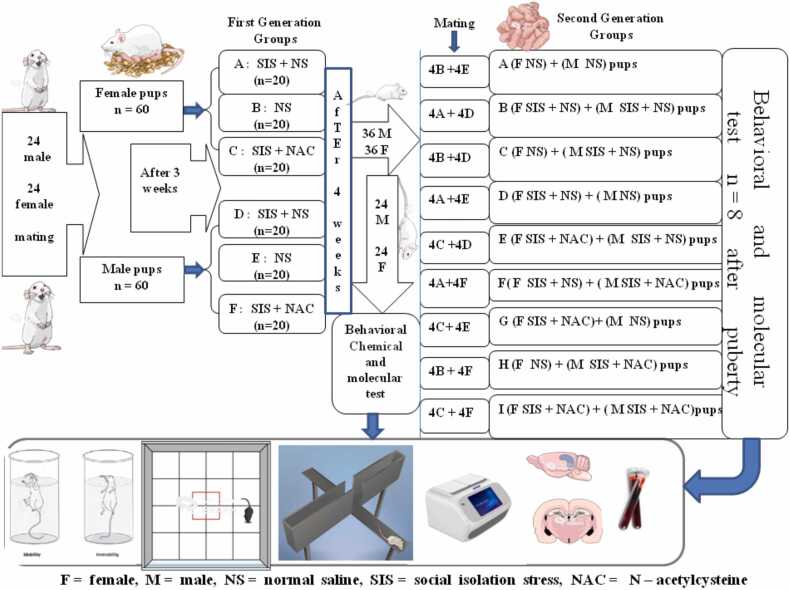
Schematic of the study design.

After behavioral tests, the mice were anesthetized with 100 mg/kg ketamine and 10 mg/kg xylazine intraperitoneally, and after decapitation, the brain and hippocampus were removed (David et al., 2022) (Fig. 1).

The order of the experiments was as follows:

First, behavioral tests were performed in the order of open field, EPM, forced swimming, and then the mouse were anesthetized and a blood sample was taken. The head was cut off and the brain was removed and kept at a temperature of minus 80 degrees Celsius until the experiments were performed.

The first generation was divided into six groups A, B, C, D, E, F and the second generation was born from the first generation by placing four male and four female mice as shown in the figure above. The second generation was grouped into groups A, B, C, D, E, F, G, H, I.

### 2.3 SIS paradigm

The mice pups lived with their mothers for three weeks after birth. After three weeks, each mouse was housed individually in a 24 × 17 × 12 cm Plexiglas box for four weeks, and its social contact with other mice and the surrounding environment was cut off. To minimize contact and maintain isolation, the cages of these animals were cleaned weekly by the same experimenter. All experiments were conducted between 10 am and 2 pm (Ayilara and Owoyele, 2023, Amini-Khoei et al., 2024).

### 2.4 Administration of NAC

From the end of the third week, NAC (sigma-Aldrich grade>99 %) was injected Intraperitoneal into the mice daily for 4 weeks at a dose of 150 mg/kg (Romero-Miguel et al., 2024).

### 2.5 Behavioral tests

#### 2.5.1 EPM

This plus-shaped maze has two closed arms and two open arms. Its height is 70 cm from the ground. When rodents are anxious, they enter the closed arms and stay there. A non-anxious animal spends more time in open arms to learn more about its surroundings. The longer the animal stays in the closed arm, the greater the animal's anxiety (Danduga and Kola, 2024).

#### 2.5.2 Open-field test (OFT)

OFT was performed to assess motor performance and stress-like behavior. A square fiberglass box measuring 30 × 30 cm was used. The screen was divided into 25 equal squares, 5 on each side (McIlwraith et al., 2024). After the final injection, the mice were individually placed in a corner of the box and their behavior was observed for 5 min. The box was cleaned with an alcohol-soaked cotton swab after each trial to prepare for the next mice and to ensure consistent entry and test conditions for all subjects. The OFT was performed immediately before the FST to account for ambulatory behavior. And also confirm that adjustments occurring in locomotor activity do not affect immobility time in the FST. The experiment was conducted in a quiet environment under a fluorescent lamp placed directly above the box (Asgharzadeh et al., 2021).

#### 2.5.3 Forced swimming test (FST)

We used FST as described in DJP David's original article, with some modifications. Mice were placed individually in glass cylinders (height: 25 cm, diameter: 10 cm) filled with 10 cm of water at a temperature of 23–25 °C for 6 min. A mouse is immobile if it floats vertically and only slightly moves to keep its head above water. Mice were tested and the immobility time was recorded in the last 4 min of the 6 min, after 2 min of habituation. Experiments were performed by well-trained experimenters who were blinded to assigned treatment (Amada et al., 2023, David et al., 2003).

### 2.6 Study of gene expression

#### 2.6.1 Quantitative real-time polymerase chain reaction (qRT-PCR)

The mouse brain was dissected to remove the hippocampus, which was immediately frozen in liquid nitrogen and stored at −80°C until analysis. Total RNA was extracted from the hippocampus using Trizol reagent (Invitrogen, Cergy Pontoise, France). Changes in mRNA levels were assessed via qRT-PCR (Buhner et al., 2022). Specifically, 1 μg of RNA from each sample underwent reverse transcription with the Prime Script RT reagent kit (Takara Bio Inc., Otsu, Japan). qRT-PCR was conducted using SYBR Premix Ex Taq technology (Takara Bio) on a Roche photocycle (Roche Diagnostics, Mannheim, Germany). Primer sequences are listed in Table 1. B2m served as the housekeeping gene, and fold changes in the expression of each target mRNA relative to B2m were calculated using the 2-ΔΔCt formula (Arabi et al., 2021) (Table 1).

**Table 1:** The genes and primer sequences used in polymerase chain reaction (PCR) amplification.

Primer	Forward sequence	Reverse sequence
HDAC_2_	TGAGGATGAAGGTGAAGGAGGT	GCTGAGTTGTTCTGACTTGGCT
GCN_5_	ACTGGGAAAGGAGAAGGGCAAG	TTCAGGTCAATGGGGAAACGGA
B2m	AGATGTCTCGCTCCGTGG	TGAATCTTTGGAGTACGCTGG

Abbreviations: HDAC_2_, Histonedeacetylase 2, GCN_5_, general control non- derepressible 5, B2M, Beta-2-microglobulin

### 2.7 Histopathology

After behavioral tests, mice were anesthetized with 100 mg/kg ketamine and 10 mg/kg xylazine (David et al., 2022). Cardiac perfusion was performed with 0.9 % normal saline and then with 4 % paraformaldehyde in 0.1 ml of cold phosphate buffer (pH = 7.5), and then, the brain was dissected out. After fixation, brain tissues were immersed in 10 % formalin. Then, 5μm sections were taken from the brains. The 5 sections taken from each brain were deparaffinized and stained with H&E staining. Histological analysis was performed under a light microscope, and then, images were displayed by embedding a digital camera attached to a computer monitor. Three fields were selected from each slide, and the density of dark neurons and natural neurons within the pyramidal layer of the CA_1_ and CA_3_ region was estimated. Three fields were selected from each slide, and the pathologist determined the diameter of the CA1, and CA3 layers using Image J software (Zhou et al., 2024).

### 2.8 Statistical analysis

Graph Pad Prism software (Version 8) was used for data analysis. Kolmogorov–Smirnov test was applied to evaluate the normal distribution of data. Using the Brown-Forsythe test, the homogeneity of variances has been checked. Statistical data analysis was conducted through a one-way analysis of variance (ANOVA), followed by Tukey’s multiple comparison test. Results were deemed statistically significant at p < 0.05.

## 3 Results

### 3.1 The effect of NAC on the number of entries in the open arm in the EPM

**Fig. 2:**
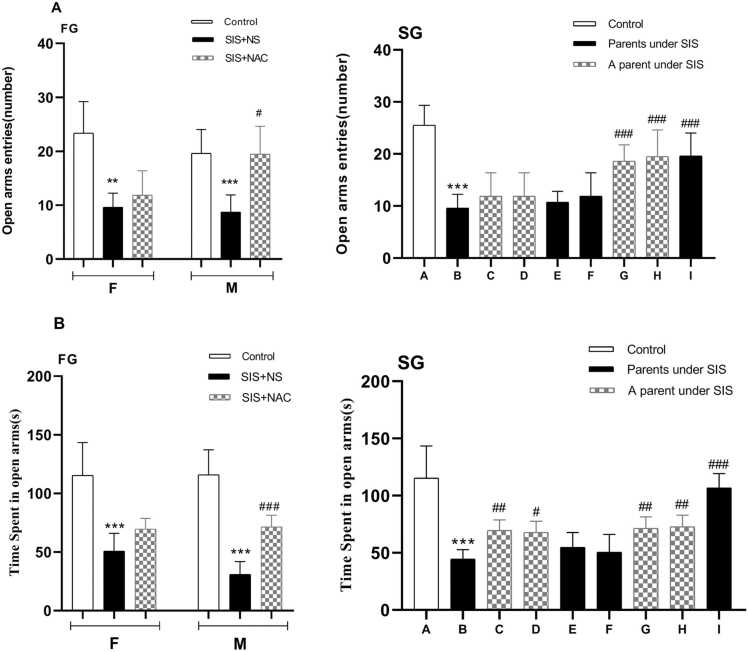
The effect of N-acetylcysteine and SIS on the frequency of entering (A) and staying time in the open arm (B) of the EPM. Values are shown as mean ± standard error. *p < 0.05, **P < 0.01, ***P < 0.001 In comparison with the control group and #P < 0.05, ##P < 0.01, ###P < 0.001 in comparison with the B group in second generation. FG=first generation, SG= second generation, F=female, M=male, NS= normal saline, SIS= Social isolation stress, NAC= N acetyl cysteine. A= Pups whose parents received NS, B= Pups whose parents subjected to SIS and received NS, C= Pups whose parents received NS and whose fathers subjected to SIS, D= Pups whose parents received NS and whose mothers subjected to SIS, E = Pups whose parents were subjected to SIS and whose mothers received NAC and whose fathers received NS, F= Pups whose parents were subjected to SIS and whose fathers received NAC and whose mothers received NS, G= Pups whose mothers subjected to SIS and received NAC and whose fathers received NS, H= Pups whose mothers received NS and whose fathers subjected to SIS and received NAC, I= Pups whose parents subjected to SIS and received NAC.

As shown in Fig. 2A, NAC significantly affected the number of entries into the open arm of the elevated plus maze (EPM) test. One-way analysis of variance showed significant differences in open-arm entries between groups. Tukey's post hoc test showed that the SIS group receiving normal saline had less access to the open arm than the control group (P < 0.01 for females, P < 0.001 for males). On the contrary, first-generation mice male SIS receiving NAC showed a significant increase in open arm entries compared to the SIS group (P < 0.001). Also, the second-generation pups whose parents received NAC showed a significant increase in open arm entries compared to the SIS group (P < 0.001). In addition, second-generation infants exposed to SIS had fewer entries into the open arm than the control group (P < 0.001). Overall, NAC treatment increased the number of entries into the open arm compared to the SIS group (P < 0.001).

### 3.2 The effect of NAC on the duration of staying in the open arm in the EPM

The findings regarding the effect of NAC on the length of stay in the open arm in the elevated plus maze test are shown in the graph of Fig. 2B. One-way analysis of variance showed a significant difference in the duration of staying in the open arm between different groups. Tukey's post hoc test showed a significant decrease in the time spent in the open arm for the SIS group receiving normal saline compared to the control group (P < 0.001). Also, the duration of staying in the open arm in males of the SIS group receiving NAC of the first generation compared to the SIS group increased significantly (P < 0.001), as well as the pups of the second generation whose parents received NAC compared to the SIS group. The duration of their stay in the arm increased significantly (P < 0.001). In the second generation of infants with parents exposed to SIS, the time spent in the open arm was significantly less than in the control group (P < 0.001). With the group receiving parents SIS (P < 0.001).

### 3.3 The effect of NAC on the number of crossings in OFT

**Fig. 3:**
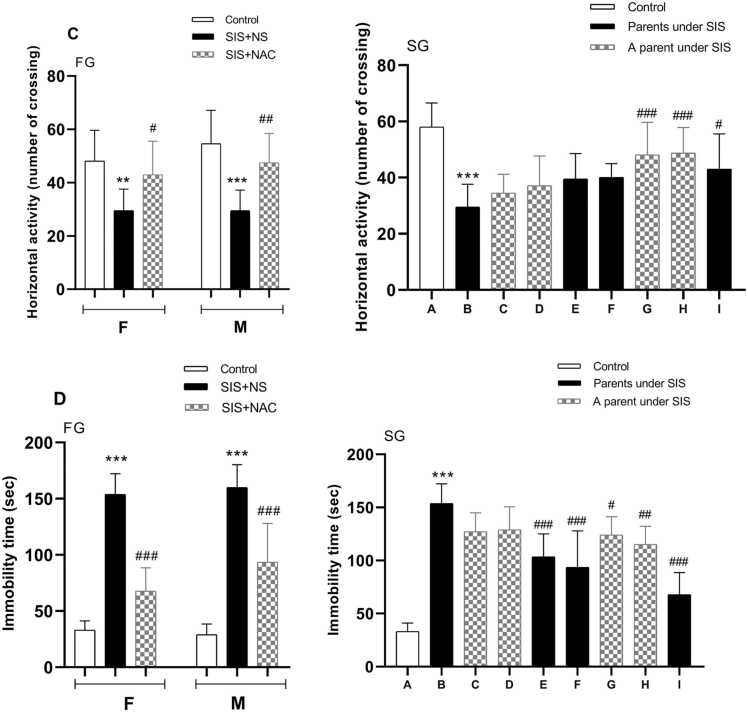
The effect of N-acetylcysteine and SIS on the open field (C) and forced swimming test (D). Values are shown as mean ± standard error. ***P < 0.001 In comparison with the control group and #P < 0.05, ##P < 0.01, ###P < 0.001 in comparison with the B group in second generation. FG=first generation, SG= second generation, F=female, M=male, NS= normal saline, SIS= Social isolation stress, NAC= N acetylcysteine. A= Pups whose parents received NS, B= Pups whose parents subjected to SIS and received NS, C= Pups whose parents received NS and whose fathers subjected to SIS, D= Pups whose parents received NS and whose mothers subjected to SIS, E = Pups whose parents were subjected to SIS and whose mothers received NAC and whose fathers received NS, F= Pups whose parents were subjected to SIS and whose fathers received NAC and whose mothers received NS, G= Pups whose mothers subjected to SIS and received NAC and whose fathers received NS, H= Pups whose mothers received NS and whose fathers subjected to SIS and received NAC, **I**= Pups whose parents subjected to SIS and received NAC.

The results regarding the effect of NAC on the number of passages in the OFT are shown in the graph (Fig. 3C) Post-test analysis confirmed that OFT crossing was significantly reduced in the SIS group receiving normal saline compared to the control group receiving normal saline(for female, P < 0.01 and male, P < 0.001). Also, the number of crossing in males of the SIS group receiving first-generation NAC and second-generation pups whose parents received NAC significantly increased compared to the SIS group (P < 0.001). In the second generation of infants whose parents were both exposed to SIS, crossing in the OFT was significantly reduced compared to the control group (P < 0.001). NAC increased the crossing rate in the OFT in the groups receiving this substance compared to the group receiving parental SIS (P < 0.001).

### 3.4 The effect of NAC on the immobility time in FST

The effects of NAC on immobility time in the FST are presented in Fig. 3D. Data analysis showed that there was a significant difference in immobility time between different groups. that the immobility time in FST in the SIS group receiving normal saline increased significantly compared to the control group receiving normal saline (P < 0.001). Also, Immobility time in males of the SIS group receiving first-generation NAC and second-generation pups from NAC-treated parents was significantly reduced compared to the SIS group (P < 0.001).In the second generation of infants whose parents were both exposed to SIS, immobility time in the FST was significantly increased compared to the control group (P < 0.001). NAC decreased immobility time in the groups receiving this substance compared to the group receiving parental SIS (P < 0.001).

### 3.5 Comparison of HDAC_2_ gene expression in the studied groups

**Fig. 4:**
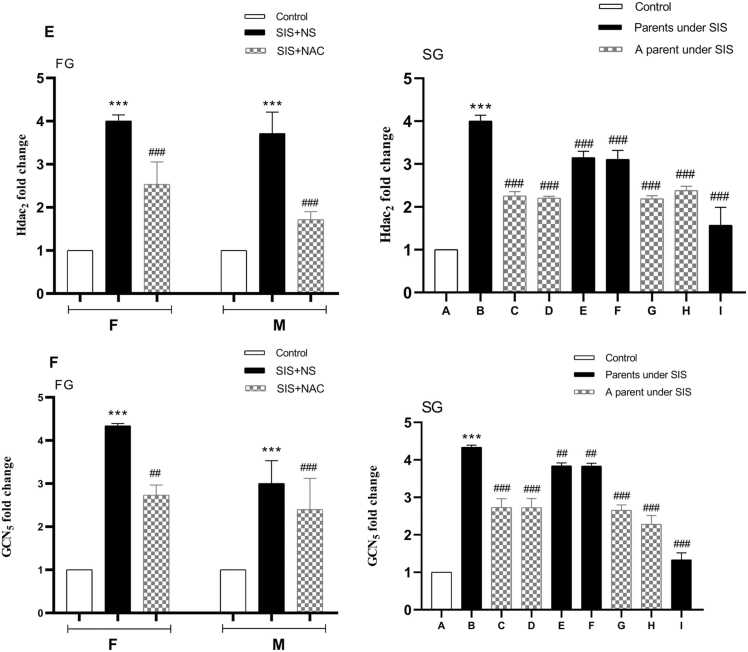
The effect of N-acetylcysteineand SIS on Hdac2 (E) and GCN5 (F) genes expression. Values are shown as mean ± standard error. **p < 0.01, ***P < 0.001 In comparison with the control group and ##P < 0.01, ###P < 0.001 in comparison with the B group in second generation. FG=first generation, SG= second generation, F=female, M=male, NS= normal saline, SIS= Social isolation stress, NAC= N acetyl cysteine. A= Pups whose parents received NS, B= Pups whose parents subjected to SIS and received NS, C= Pups whose parents received NS and whose fathers subjected to SIS, D= Pups whose parents received NS and whose mothers subjected to SIS, E = Pups whose parents were subjected to SIS and whose mothers received NAC and whose fathers received NS, F= Pups whose parents were subjected to SIS and whose fathers received NAC and whose mothers received NS, G= Pups whose mothers subjected to SIS and received NAC and whose fathers received NS, H= Pups whose mothers received NS and whose fathers subjected to SIS and received NAC, I= Pups whose parents subjected to SIS and received NAC.

According to the results of ANONA analysis and post hoc test of Tukey's test, the results of this study showed that the expression level of the HDAC_2_ gene has a significant difference between different groups (P < 0.001). So, the SIS group receiving normal saline compared to the control group increased significantly (P < 0.001). Also, there was a significant decrease in the SIS group receiving NAC compared to the SIS group receiving normal saline (P < 0.001). The expression of the HDAC_2_ gene in the second generation in the group where parents underwent SIS group receiving normal saline significantly increased compared to the control group (P < 0.001). Gene expression in the groups receiving NAC was significantly reduced compared to the group where parents underwent SIS receiving normal saline (P < 0.001) (Fig. 4E).

### 3.6 Comparison of GCN_5_ gene expression in the studied groups

The study results indicate significant differences in GCN_5_ gene expression among groups (P < 0.001). The SIS group receiving normal saline showed a marked increase in GCN_5_ expression compared to the control group (P < 0.001). Furthermore, GCN_5_ expression decreased significantly in the SIS group receiving NAC compared to the SIS group receiving normal saline (P < 0.001). In the second generation, the offspring of parents receiving normal saline SIS exhibited significantly higher GCN_5_ expression compared to the control group (P < 0.001). Additionally, GCN_5_ expression in NAC-treated groups was significantly lower than in the SIS group receiving normal saline (P < 0.001) (Fig. 4F).

### 3.7 The results of histological studies

#### 3.7.1 Estimation of the diameter of the CA_1_ region

**Fig. 5:**
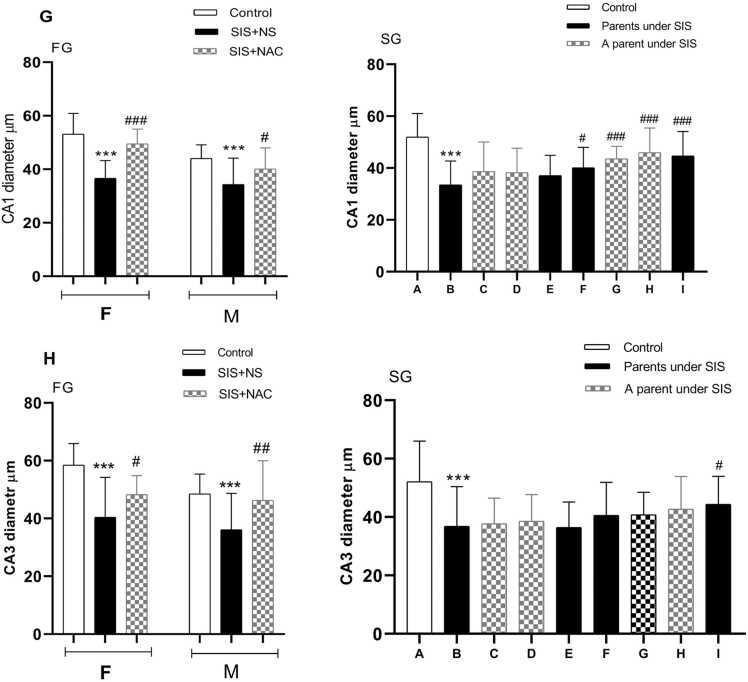
The effect of N-acetylcysteine and SIS on the CA1 (G), CA3 (H) diameter. Values are shown as mean ± standard error. *P < 0.05, **P < 0.01 In comparison with the control group and #P < 0.05, in comparison with the B group in second generation. FG=first generation, SG= second generation, F=female, M=male, NS= normal saline, SIS= Social isolation stress, NAC= N acetyl cysteine. A= Pups whose parents received NS, B= Pups whose parents subjected to SIS and received NS, C= Pups whose parents received NS and whose fathers subjected to SIS, D= Pups whose parents received NS and whose mothers subjected to SIS, E = Pups whose parents were subjected to SIS and whose mothers received NAC and whose fathers received NS, F= Pups whose parents were subjected to SIS and whose fathers received NAC and whose mothers received NS, G= Pups whose mothers subjected to SIS and received NAC and whose fathers received NS, H= Pups whose mothers received NS and whose fathers subjected to SIS and received NAC, I= Pups whose parents subjected to SIS and received NAC.

**Fig. 6:**
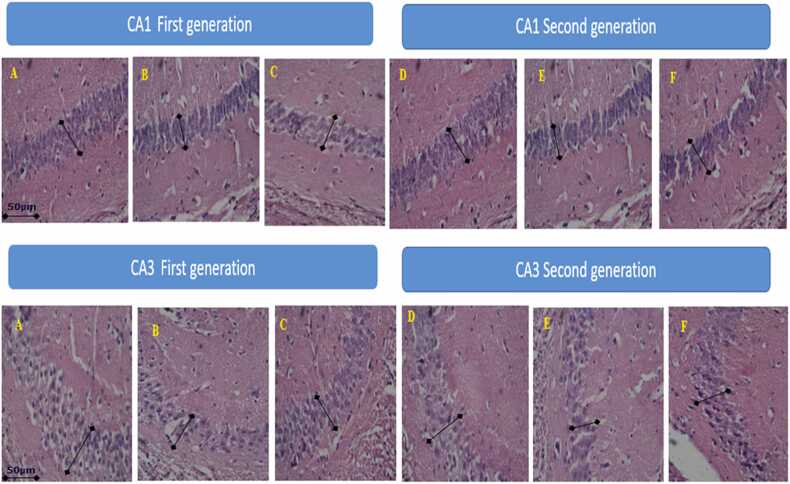
It shows the characteristics of the diameter of the neurons in the CA_1_ and CA_3_ regions of the hippocampal pyramidal region of the first generation. (A) Control, (B) SIS, (C) SIS + NAC and similar features in the second generation: (D) Control, (E) SIS, (F) SIS + NAC, SIS= Social isolation stress, NAC= N acetyl cysteine.

One-way analysis of variance showed that the diameter of the CA_1_ region decreased in the SIS group compared to the control group (P < 0.001). Nevertheless, treatment with NAC prevented the decrease in the diameter of the CA_1_ region, so the diameter of the CA_1_ region in this group did not show a difference from the control group. In addition, treatment with NAC showed a significant increase compared to the SIS group that received normal saline (P < 0.05). The diameter of the CA_1_ region of the hippocampus in the second generation of mice whose parents were subjected to SIS receiving normal saline was significantly reduced compared to the control group (P < 0.001). The diameter of this area was significantly increased in the groups that received NAC compared to the group whose parents received SIS receiving normal saline (P < 0.001) (Figs. 5G and 6).

#### 3.7.2 Estimation of the diameter of the CA_3_ region

The effect of NAC administration on the diameter of the CA_3_ region is shown in graph Figs. 5H and 6. Compared to the control group, the diameter of the CA_3_ in the SIS group receiving normal saline was significantly lower (P < 0.001). The diameter of the CA_3_ region in the hippocampus in the NAC group showed a significant difference from the control group (P < 0.05). The diameter of the CA_3_ area of the hippocampus in the second generation of the offspring of mice whose parents were subjected to the SIS group receiving normal saline was significantly reduced compared to the control group (P < 0.001). The diameter of this area increased significantly in the groups that received NAC compared to the group that parents underwent SIS group receiving normal saline (P < 0.01).

## 4 Discussion

The aim of this study is to the effect of NAC through the modulation of HDAC_2_ and GCN_5_ in the hippocampus and investigate behavioral disorders in the first and second generation of SIS mice. The results of this study showed that SIS in the first and second generations causes psycho-behavioral disorders. In this study, it was found that NAC reduces behavioral disorders in the first and second generations. The results showed that NAC decreased the expression of HDAC_2_ and GCN_5_ genes in the first and second generations and increased the diameter of the CA_1_ and CA_3_ areas of the hippocampus.

SIS causes anxiety-like behaviors in early life and emotional changes as well as a persistent state of anxiety (Amiri et al., 2015). We observed in the first generation that the number and time of staying in the open arm of EPM is reduced. Interestingly, a decrease in the number of admissions and length of stay in the open arm was also observed in the second generation. This confirms the role of epigenetic factors in the inheritance of mental disorders from the first to the second generation (Kosten and Nielsen, 2014).

The results of this study are consistent with previous studies that reported that SIS is capable of stimulating depressive behaviors in mice (Marco et al., 2015). We showed that SIS mice had increased immobility time in the FST compared to control animals. Increased immobility time in FST indicates behavioral frustration in mice. Our findings showed that NAC treatment significantly reduced immobility time in the FST, suggesting that NAC has antidepressant effects (Nouri et al., 2020).

OFT is performed to assess motor activity in different conditions. The OFT was performed immediately before the FST to confirm that adjustments occurring in locomotor activity did not affect immobility time in the FST. The OFT is also a valid tool for assessing anxiety-like behaviors, in which the time spent in the central region of the device is considered as a parameter of anxiety (Amini-Khoei et al., 2024). SIS significantly reduced the time spent in the central region in the first generation. Also, SIS significantly reduced the time spent in the central region in the second generation as well. Which makes the role of epigenetic factors in the inheritance of mental disorders from the first to the second generation more colorful. Amiri et al. (2015) showed an increase in immobility time in the FST in SIS mice was indicative of depressive behavior, which was observed in the results of this study in both generations (Amiri et al., 2015).

In this study and in confirmation of other studies, the expression level of HDAC_2_ and GCN_5_ genes showed a significant increase in mice under early-life stress (Qi et al., 2021). Histone deacetylase and acetyltransferase genes show their epigenetic role by moving acetyl groups on DNA (Wei et al., 2020). In the second-generation SIS mice, the expression of HDAC_2_ and GCN_5_ genes showed a significant increase in the offspring of mice exposed to SIS compared to mice with healthy parents. These findings are consistent with previous research (Yang et al., 2024).

The hippocampus is a part of the limbic system that plays an important role in the pathophysiology of depression. Exposure of mice to chronic stress increases the neurodegeneration of the CA_1_ and CA_3_ areas of the hippocampus (Parashar et al., 2017). According to the studies of Anjomshoa et al., chronic stress is associated with neurodegeneration in the hippocampal pyramidal region. They found that chronic stress reduces the diameter of the CA_3_ region (Anjomshoa et al., 2020). In Pan et al.'s study, it was observed that SIS causes a decrease in the diameter of neurons in CA_1_ and CA_3_ regions, (Pan et al., 2022). In the present study, SIS reduced the diameter of neurons in the CA_1_ and CA_3_ regions of the hippocampus. This happened in the second generation as well.

NAC is a substance with antioxidant effects (Siswanto and Mindiroeseno, 2023). Investigating the mechanisms behind the effects of NAC could reveal potential therapeutic strategies for psychobehavioral disorders (Poladian et al., 2023). The cellular mechanisms by which NAC exerts its protective effects may involve modulation of signaling pathways associated with oxidative stress and inflammation. For example, recent studies have shown that NAC increases the activity of glutathione, a key endogenous antioxidant, thereby reducing oxidative damage in the brain (Caridade-Silva et al., 2023). In the present study, it was proved that NAC, as a strong antioxidant in the brain, reduces the expression of HDAC_2_ and GCN_5_ genes. The role of NAC was also evident in the hippocampus, as the diameter of neurons in this region was significantly increased compared to SIS subjects who received saline.

Past research has emphasized the importance of timing in pharmacological interventions, particularly during developmental stages when neural plasticity increases (Curpan et al., 2021). It is also pertinent to note the gender differences that may pertain to the susceptibility to stress-induced behavioral changes and response to treatment (Shirenova et al., 2023). Emerging evidence suggests that male and female rodents may exhibit divergent responses to SIS and subsequent antioxidant treatments, reflecting the need for gender-disaggregated analyses in preclinical and clinical studies (Benvenuti et al., 2024). In this study, we do not observe differences between male and female mice.

previously intersection of genetic predisposition and epigenetic modifications induced by environmental stressors has been determined (Panariello et al., 2022). Epigenetic alterations triggered by early-life stress could have profound implications for understanding the heritability of mental disorders (Alameda et al., 2022). The interplay between environmental factors and genetic predisposition necessitates a comprehensive approach to studying mental health outcomes across generations (Nigg, 2023). The intersection of genetic predisposition and epigenetic changes induced by environmental stressors has already been identified (Panariello et al., 2022). Epigenetic changes induced by stress in early life can have profound implications for understanding the heritability of mental disorders (Alameda et al., 2022). The interplay between environmental factors and genetic predisposition necessitates a comprehensive approach to studying mental health outcomes across generations (Nigg, 2023). In this study, we showed that the second generation like the first generation in behavioral tests, EPM decreased the number and duration of staying in the open arm and increased the immobility time in the FST test, which indicates behavioral frustration in mice. And in the OFT test, it also reduced the time spent in the central region. The expression of HDAC_2_ and GCN_5_ genes showed an increase and decrease in the diameter of neurons in the CA_1_ and CA_3_ regions of the hippocampus. This confirms the role of epigenetic factors in the inheritance of mental disorders from the first to the second generation.

In conclusion, the findings of this study N-acetylcysteine mitigating the adverse effects of SIS-induced mental disorders. The potential for these compounds to break the cycle of intergenerational transmission of mental health issues warrants further research and consideration in therapeutic contexts. With increasing awareness of the multifactorial nature of mental health, integrative strategies combining pharmacological interventions with lifestyle modifications, including stress management techniques and nutritional support, could enhance the overall efficacy of treatments aimed at improving mental well-being. Continued exploration in this field is essential, particularly as we strive to address the growing public health concerns associated with mental disorders in contemporary society.

## 5 Conclusion

In conclusion, SIS is associated with behavioral disorders in first and second-generation mice these behaviors are associated with increased HDAC_2_ and GCN_5_, as well as decreased diameter and number of neurons in the hippocampus. NAC reversed the negative effect of SIS in the first and second generations partially decreasing HDAC_2_ and GCN_5_, as well as improving hippocampus structure

## Authors' Contribution

All authors have approved the manuscript.

## CRediT authorship contribution statement

**asgharzadeh najmeh:** Writing – review & editing, Writing – original draft, Software, Formal analysis, Data curation, Conceptualization. **shahrani mehrdad:** Validation, Methodology, Investigation, Formal analysis, Conceptualization. **Amini-Khoei Hossein:** Writing – review & editing, Supervision, Investigation, Formal analysis. **Yazdanpanahi Nasrin:** Visualization, Formal analysis. **nori ali:** Methodology, Formal analysis, Conceptualization.

## Declaration of Competing Interest

The authors declare that they have no conflicts of interest.
